# Comparative Analysis of Physical and Mechanical Properties of Acrylic Resins for Interim Fixed Prostheses Under Thermocycling Aging

**DOI:** 10.3390/bioengineering13050510

**Published:** 2026-04-28

**Authors:** Emily Vivianne Freitas da Silva, Carolina Lucena e Ortiz, Marina Silveira Gomes, Wendy Julliet Alvarado Baldeon Condor, Karina Felix Santos, Savio José Cardoso Bezerra, Paulo Francisco Cesar, Natalia Almeida Bastos-Bitencourt, Sandro Basso Bitencourt, Blanca Liliana Torres Léon

**Affiliations:** 1 Department of Prosthodontics, São Paulo Dental School, University of São Paulo (USP), São Paulo 05508-000, São Paulo, Brazil; carolinaortiz@usp.br; 2 Bahia Dental School, Federal University of Bahia (UFBA), Salvador 40110-150, Bahia, Brazil; marinasgomes1@hotmail.com (M.S.G.); blalitole@hotmail.com (B.L.T.L.); 3 Bahia Medicine and Public Health School, Salvador 40285-001, Bahia, Brazil; wendyjuliett@outlook.com; 4 Department of Biomaterials and Oral Biology, São Paulo Dental School, University of São Paulo (USP), São Paulo 05508-000, São Paulo, Brazil; karina.felix.santos@usp.br (K.F.S.); paulofc@usp.br (P.F.C.); 5 Department of Dentistry, São Paulo Dental School, University of São Paulo (USP), São Paulo 05508-000, São Paulo, Brazil; saviobezerra@usp.br; 6 Department of Comprehensive Dentistry, University of Louisville School of Dentistry, Louisville, KY 40202, USA; natalia.bastosbitencourt@louisville.edu; 7 Department of Rehabilitative & Reconstructive Dentistry, University of Louisville School of Dentistry, Louisville, KY 40202, USA; sandro.bitencourt@louisville.edu

**Keywords:** acrylic resins, denture bases, flexural strength

## Abstract

This study evaluated the physical and mechanical properties of acrylic resins used for interim fixed prostheses, with and without metal reinforcement, before and after aging. A total of 138 samples were divided into three groups: VIPI + Wire (control), VIPI, and Diamond D. Samples were assessed for microhardness, porosity, roughness, and flexural strength. Aging was simulated using 500 thermocycling cycles at 5 and 55 ± 1 °C. Data were analyzed using ANOVA and Tukey’s test. Group Diamond D did not fracture during flexural testing, but it exhibited significantly lower microhardness at both baseline and after aging. Before aging, Group Diamond D had higher roughness than Group VIPI, which exhibited greater porosity. Aging increased the microhardness of Group VIPI and the roughness of Group Diamond D. The percentage of porosity decreased significantly for Groups VIPI + Wire and VIPI, and pore size was reduced in all groups. Based on the results obtained from Diamond D material, this resin does not meet the required properties for the proposed indication for temporary fixed prostheses, whereas VIPI with reinforcement showed superior properties and greater stability after aging.

## 1. Introduction

To rehabilitate an edentulous patient, a conventional complete denture made of acrylic resin (polymethyl methacrylate) is indicated [1]. Another treatment alternative is the implant-supported fixed complete denture, which offers an excellent strategy for restoring esthetics, function, and mastication. Compared with conventional complete dentures, these prostheses offer greater stability and retention and provide greater satisfaction to wearers, as demonstrated in several clinical studies [2,3,4]. They are usually screwed in place, making them reversible, so they can be removed to retighten or replace screws, substitute fractured components, clean, and assess peri-implant tissues [5,6].

During the rehabilitation process with implant-supported fixed complete dentures, it may be necessary to use conventional dentures between implant placement and installation of the definitive prosthesis. This situation commonly occurs when not all implants can support immediate load after surgery, requiring 3 to 6 months for osseointegration [7]. However, implants with adequate length and initial bone stability can support a provisional implant-supported fixed denture for immediate loading, reestablishing esthetics and function, while allowing osseointegration of the remaining implants. This provides stability and comfort, supporting both esthetic and masticatory functions [8].

Implant-supported fixed provisional complete dentures are transitional prostheses used temporarily before definitive rehabilitation. They must have satisfactory strength and aesthetics and provide proper cleansability [9]. These prostheses are usually fabricated using an acrylic resin structure with artificial teeth. Additionally, they incorporate provisional prosthetic components (such as titanium cylinders) that support the prosthesis. Metallic reinforcements can be added to improve strength and stiffness [10]. Unlike the definitive prosthesis, which generally incorporates a metal bar cast into its internal structure, the metal reinforcement of the provisional prosthesis often uses materials such as thick orthodontic wire [11].

Due to high masticatory distress and accidental damage, fracture is a common complaint among denture wearers [12,13]. Provisional implant-supported fixed dentures have lower resistance to occlusal forces than definitive dentures due to the absence of metal internal reinforcement [14,15]. This reinforcement compensates for the low flexural strength and high fracture risk of PMMA-based prostheses [11,16]. To optimize fracture resistance, manufacturers can incorporate reinforcements such as copolymers or fibers [16]. In the dental market, one brand stands out due to its ultra-impact thermosetting acrylic resin composition. According to the manufacturer, this resin eliminates the need for metal reinforcements and meshes, thanks to its high impact strength and exceptional flexural strength [17,18,19].

During use, but especially during cleaning, the prosthesis is subjected to abrasion forces that may result in scratches and plastic deformation. Lower microhardness indicates a weaker surface, which is more susceptible to damage [20].

Microbial contamination can compromise the integrity of biological tissues (bone and soft tissue) surrounding osseointegrated implants [21]. Therefore, ensuring the prosthesis maintains a smooth surface, thereby reducing the space available for microbial deposits, is crucial for osseointegration. Surface porosity and roughness significantly influence smoothness.

Temperature variation and oral fluid absorption in denture polymers can affect the prosthesis’ physical properties [22]. Thermocycling is commonly used in vitro to simulate temperature changes in the mouth [23]. One of the most widely used techniques in scientific research is thermocycling, which simulates the extreme temperatures to which materials are exposed in the oral environment [24]. According to Carreira et al., materials tend to expand when heated and shrink when cooled. These temperature variations can generate mechanical stresses at the interface between the organic resin matrix and inorganic filler particles due to differences in their thermal expansion coefficients. This can lead to microleakages and fatigue fractures associated with the hydrolytic degradation of the polymer matrix and filler particles [24,25,26]. Aging and temperature variation can therefore influence resin properties [22,23].

Given the various reinforcement techniques available to strengthen PMMA-based resins and the different commercial brands on the market, this study compared the flexural strength, microhardness, roughness, and porosity of two PMMA-based resins. VIPI Cril Plus resin is widely used in dental practice and well-documented in the literature [27,28,29,30], making it the control in this study. The purpose was to determine whether Diamond D resin could be used as the base for a provisional implant-supported fixed full-arch denture without internal metallic reinforcement, as proposed by the manufacturer, while still providing adequate flexural strength and surface smoothness. The null hypothesis was that no differences would be observed between the resin groups, regardless of internal metal reinforcement or aging.

## 2. Materials and Methods

**Figure 1:**
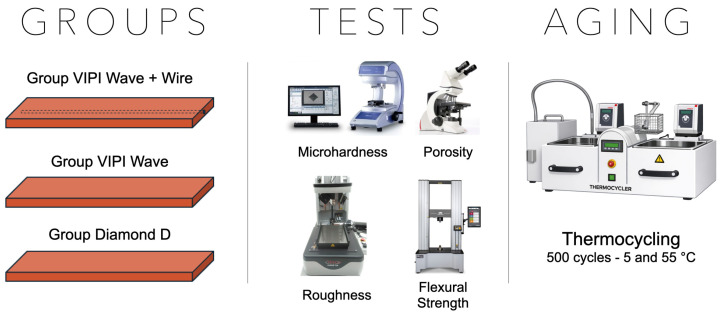
Experimental workflow used in the study.

The experimental design of the present study involved the preparation of 138 acrylic resin samples, divided into 3 groups: Group VIPI + Wire (VIPI Cril Plus with metal reinforcement, control), Group VIPI (VIPI Cril Plus without reinforcement), and Group Diamond D (Diamond D without reinforcement). The VIPI + Wire was considered the control group, to replicate the clinical scenario, as these provisional implant-supported fixed full-arch dentures often need a metal reinforcement. The samples were evaluated for microhardness, porosity, roughness, and flexural strength before and after 500 thermocycling cycles (Figure 1). The sample size was determined based on a pilot study performed for roughness and flexural strength tests. A power analysis was conducted with a level of significance of 0.05, 80% power, and a medium effect size (GPower 3.1 software). The power analysis indicated that 8 samples were required for the roughness test and 15 for the flexural strength test. Thus, 10 and 18 samples were used, respectively. For roughness and porosity tests, the same samples were used before and after accelerated aging (*n* = 10/group). For microhardness and flexural strength analyses, half of the samples were tested before aging (*n* = 18/group), and the other half after aging (*n* = 18/group).

Samples were prepared according to a previously established protocol using a metal mold with dimensions of 67 mm in length, 12 mm in width, and 3 mm in height, in accordance with ANSI/ADA specification No. 12 [31]. VIPI Wave acrylic resin samples (VIPI^®^ Indústria, Comércio, Exportação e Importação de Produtos Odontológicos, Pirassununga, Brazil) were prepared following the manufacturer’s instructions. For the VIPI Wave samples with orthodontic wires, a channel-shaped cavity was created with a Carbide spherical drill (KG Sorense, Cotia, Brazil) to insert a 0.8 mm metal wire (Morelli Ortodontia, Sorocaba, Brazil). After inserting the wire, additional resin was applied, and the samples were pressed again [32]. Polymerization was carried out in a microwave oven (Electrolux S/A, MEF41-31 L, 1000 Watts, Curitiba, Brazil) with an initial cycle at 20% power for 20 min and a final cycle at 60% power for 5 min [33].

For the Diamond D resin samples, preparation followed the manufacturer’s recommendations. Polymerization was performed in the same microwave at 90% power for 4 min. The flasks were cooled slowly in water for 24 h, after which the samples were removed and stored [34,35].

The specimens were finished with aluminum oxide water sandpaper (Erios Equipamentos LTDA, São Paulo, Brazil) in a decreasing grit sequence (600, 800, 1200, 1500, 4000) under refrigeration, using an Arotec Model APL-4 horizontal polisher (Arotec Indústria e Comércio Ltda, Cotia, Brazil). Polishing was carried out with felt disks (American Burrs, Palhoça, Brazil) and polishing paste (KOTA Indústria e Comércio LTDA, São Paulo, Brazil) [36]. Sample thickness was measured using a digital caliper (500-171-20B, Mitutoyo, Kawasaki, Japan). The samples were stored at room temperature (23 ± 2 °C) in distilled water for 24 h prior to testing [37].

Knoop microhardness was measured using a microhardness tester (Shimadzu, Kyoto, Japan) according to ASTM E384 [38]. Three indentations were made on each sample using a 25 g load for 10 s. For surface porosity evaluation, optical microscopy (DM RVE, Wetzlar, Germany) was used, and pore area fraction was determined from 5 optical micrographs for each group (Leica QWin, Wetzlar, Germany) [39]. Surface roughness was assessed with a profilometer (Dektak d-150; Veeco, Plainview, NY, USA). Ra values were measured with a 500 μm cut-off for 12 s, with three readings taken on each sample and the average calculated. These values were given in Ångströms (Å) and subsequently converted to nanometers (nm) [40].

Flexural strength was analyzed using a 3-point bending test (ISO 20795-1) [41] on a mechanical testing machine (EMIC DL 2000, EMIC Equipamentos e Sistemas de Ensaios Ltda, São José dos Pinhais, Brazil). Samples were placed horizontally on metal support rods 50 mm apart. The test was performed at a constant speed of 0.5 mm/min until fracture. Flexural strength (MPa) was calculated using the formula [42]: Fs = 3Pml/2bh^2^, where Pm is the maximum load, l is the distance between supports, b is the sample width, and h is its thickness. The specimens were aged in a thermocycler (Convel, São Paulo, Brazil), with immersion in distilled water in alternating 30 s baths at 5 ± 1 °C and 55 ± 1 °C, for a total of 500 cycles, simulating 6 months of clinical use [43,44].

Statistical analysis was performed using SPSS software (version 24.0, SPSS Inc., Chicago, IL, USA). The Kolmogorov–Smirnov test was used to assess normality of the data. Microhardness, roughness, and porosity data (pore size and percentage of pores per area) were analyzed using two-factor repeated measures ANOVA (factors: resin type and period), followed by the Tukey test. Flexural strength data were analyzed using two-factor ANOVA (factors: resin type and period). All analyses were performed at a 5% significance level. Generative artificial intelligence (GenAI) was used in the study design of this paper.

## 3. Results

**Figure 2:**
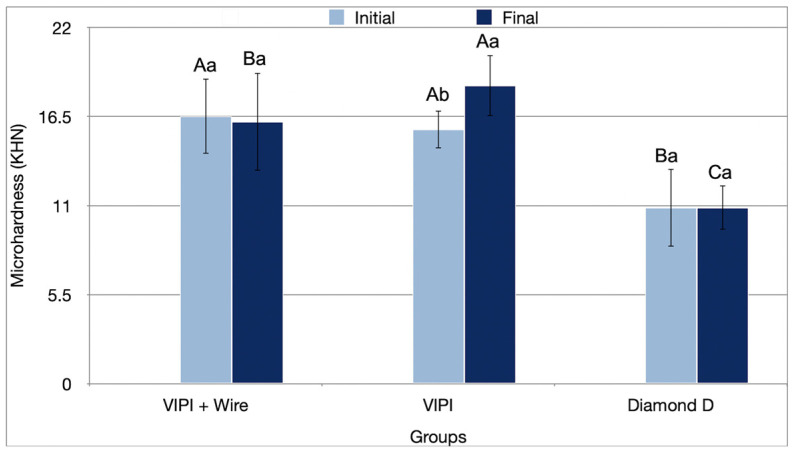
Mean values (standard deviation) of the microhardness (KHN) of the resins tested in the initial and final periods. Different uppercase letters indicate statistical differences for the same period between groups (*p* < 0.05). Different lowercase letters indicate statistical differences for the same group between periods (*p* < 0.05).

The resin type significantly influenced the microhardness results (*p* < 0.001) (Table 1). Group Diamond D showed significantly lower microhardness than the other groups in the initial (10.87 KHN, *p* < 0.001) and final periods (10.88 KHN; *p* < 0.001). Aging resulted in a significant increase in microhardness for the VIPI Group (15.70 KHN to 18.42 KHN; *p* ≤ 0.03) (Figure 2).

**Figure 3:**
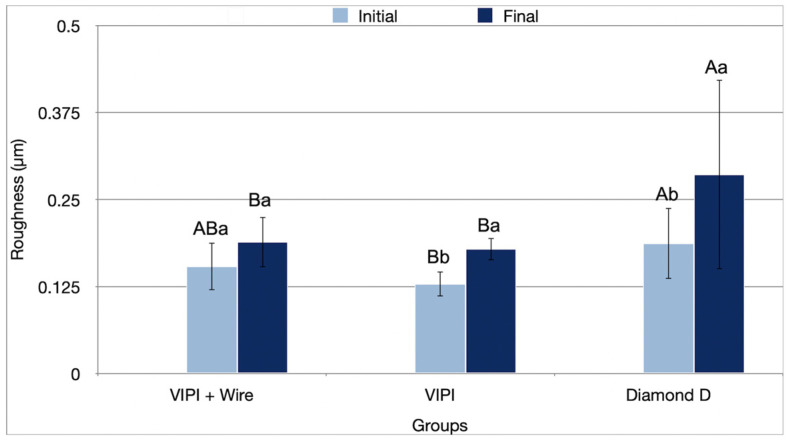
Mean values (standard deviation) of roughness (Ra) (µm) of the resins tested in the initial and final periods. Different uppercase letters indicate statistical differences for the same period between groups (*p* < 0.05). Different lowercase letters indicate statistical differences for the same group between periods (*p* < 0.05).

The interaction between resin type and period affected surface roughness (Ra) results (*p* = 0.038) (Table 1). In the initial period, Group Diamond D showed significantly higher roughness (0.187 µm) than Group VIPI (0.129 µm; *p* = 0.002). In the final period, Group Diamond D exhibited significantly higher roughness (0.286 µm; *p* ≤ 0.013) than the other groups. Roughness increased for Groups VIPI (0.129 µm to 0.179 µm; *p* = 0.008) and Diamond D (0.187 µm to 0.286 µm; *p* = 0.017) after aging (Figure 3).

**Figure 4:**
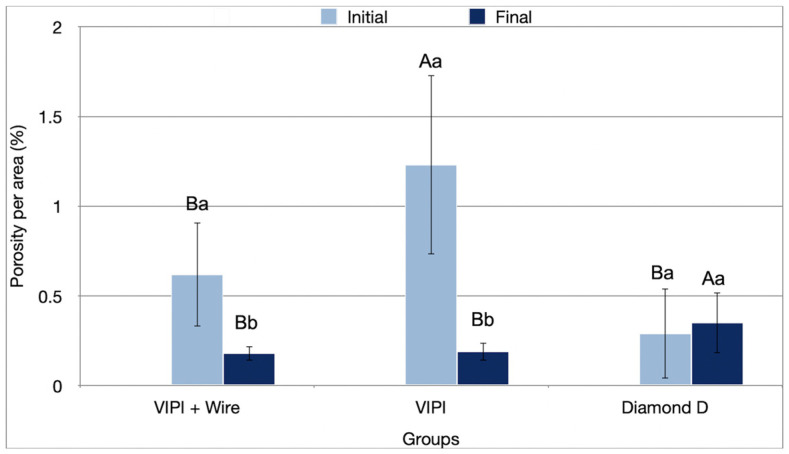
Percentage values (standard deviation) of porosity of the resins tested in the initial and final periods. Different uppercase letters indicate statistical differences for the same period between groups (*p* < 0.05). Different lowercase letters indicate statistical differences for the same group between periods (*p* < 0.05).

The interaction of resin type and period significantly impacted the porosity percentage (*p* < 0.001) (Table 1). In the initial period, Group VIPI showed a higher percentage of porosity per area (1.23%; *p* ≤ 0.001) than the other groups. By the final period, Group Diamond D showed a higher porosity percentage (1.23%; *p* ≤ 0.001) than the other groups. There was a significant reduction in the percentage of porosity in the final period for VIPI + Wire (0.62% to 0.18%; *p* = 0.003) and VIPI (1.23% to 0.19%; *p* ≤ 0.001) (Figure 4).

**Figure 5:**
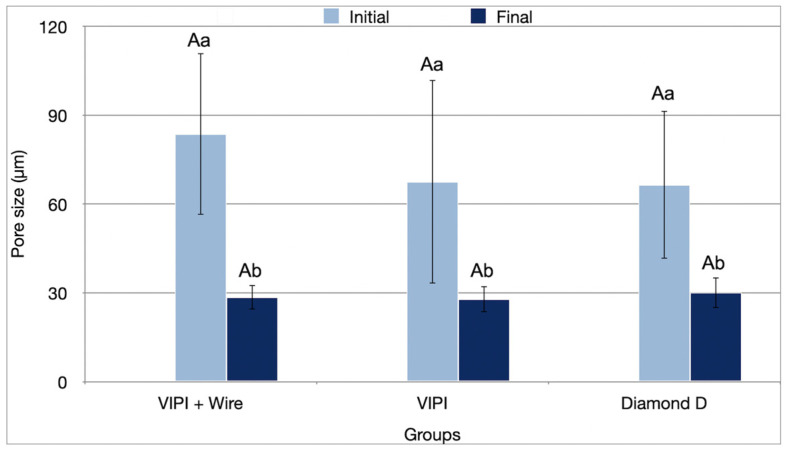
Mean pore size values (standard deviation) of the resins tested in the initial and final periods. Different uppercase letters indicate statistical differences for the same period between groups (*p* < 0.05). Different lowercase letters indicate statistical differences for the same group between periods (*p* < 0.05).

In contrast, the period factor had a significant effect on pore size (*p* < 0.001) (Table 1), with all groups showing a significant reduction in pore size after aging. Group VIPI + Wire showed a decrease from 83.65 µm to 28.50 µm (*p* ≤ 0.001), Group VIPI from 67.52 µm to 27.91 µm (*p* ≤ 0.001), and Group Diamond D from 66.48 µm to 30.05 µm (*p* ≤ 0.001) (Figure 5).

**Figure 6:**
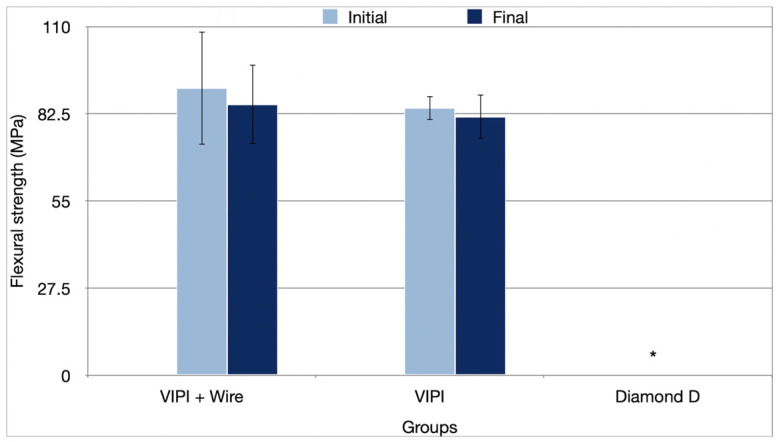
Mean values (standard deviation) of flexural strength (MPa) of the resins tested in the initial and final periods. * The test was not applicable since none of the samples fractured during testing.

None of the samples from Group Diamond D fractured during the flexural strength test, with deflections greater than 5 mm, which led to the test being canceled for this group. Therefore, the analysis was conducted only for Groups VIPI + Wire and VIPI. Neither the resin type (*p* = 0.170), nor the period (*p* = 0.296), nor their interaction (*p* = 0.737) affected the flexural strength results (Table 1), with the average values shown in Figure 6.

## 4. Discussion

During the thermally activated polymerization process of acrylic resin, polymer chains form as methyl methacrylate monomers are converted into polymers. However, residual monomer may remain in the polymerized resin as the temperature decreases. The present study standardized the sample size, thickness, shape, and polishing protocol to evaluate the physical–mechanical properties of the acrylic resins used.

Group Diamond D showed lower microhardness and higher roughness than the other groups during the initial and final periods. This result may have been attributed to the polymerization method recommended by the manufacturer and the level of residual monomer remaining in the resin [45]. Diamond D resin polymerization requires a higher temperature and a shorter polymerization time (4 min at 90% power in a 1000 W oven), which may result in more residual monomer. In contrast, VIPI Wave resin undergoes a longer polymerization cycle (20 min at 20% power, followed by 5 min at 60% power), reducing residual monomer and yielding higher microhardness and lower roughness values [46,47]. Additionally, excess heat could have compromised the material’s mechanical properties [48]. Though flexural strength tests for Group Diamond D were not feasible due to the material’s deflection greater than 5 mm, its increased roughness and lower hardness suggest the need for shorter intervals between polishing sessions in clinical practice. Additionally, alternative polymerization protocols should be investigated.

Group VIPI showed an increase in microhardness with aging. Microhardness is influenced by the degree of crosslinking and the amount of residual monomer [47]. Thermocycling may have enhanced polymerization, reducing residual monomers and thus increasing microhardness [49].

Regarding surface roughness, the ideal value for intraoral prostheses is less than 0.2 µm to minimize biofilm retention (bacteria and fungi), color alteration, and patient discomfort [50]. The polishing technique plays a significant role in surface roughness, as the speed at which the sandpaper is rotated and the pressure applied to the samples are difficult to standardize. In clinical practice, the irregular surface of complete dentures complicates polishing, potentially affecting surface roughness [51,52]. Group Diamond D showed higher roughness than the other groups, exceeding the ideal threshold after aging, with a high standard deviation. This result could be due to polymerization-induced contraction, with higher polymerization temperatures leading to greater contraction and surface roughness [53]. Additionally, this may have occurred due to polymerization at higher power in the microwave oven, with a short polymerization time, resulting in a greater number of residual monomers, which contributes to increased surface roughness [54]. It has been shown that conventional fabrication of PMMA results in greater surface roughness, which in turn leads to microcrack formation under cyclic loading, whereas digitally processed materials exhibit more homogeneous polymer structures [55,56]. Additionally, Diamond D resin includes fibers for improved strength [19], which may have contributed to the roughness [57]. After aging, roughness increased for Groups VIPI and Diamond D. This can be attributed to water absorption during thermocycling, which weakens the polymer matrix and increases surface roughness [50,58].

The porosity results were influenced by accelerated aging. Although an increase in porosity and pore size was expected, all groups showed a decrease in pore size, with Groups VIPI + Wire and VIPI also exhibiting reduced porosity percentage after aging. Despite this, porosity remained below 1.23%, which is clinically acceptable, as it is desirable to have less than 11% porosity in acrylic resin pieces [59]. In addition, the American Dental Association specifies that no bubbles or pores should be found when examined without magnification [60]. In this study, no pores or bubbles were visible without magnification. A recent systematic review comparing fabrication methods for interim restorations found that digital processing methods (3D printing and CAD/CAM) reduce internal defects and porosity, thereby directly improving the mechanical performance of these materials [61].

The Group Diamond D exhibited a high standard deviation, especially in the initial period. This may be due to the short polymerization cycle at high power, which results in rapid polymerization but may not have been sufficient to fully reduce residual monomer [48]. The high standard deviation could also result from non-homogeneous heat distribution during rapid heating.

Before aging, Groups VIPI + Wire and VIPI had greater porosity than Group Diamond D. This is likely due to the longer polymerization cycle of the VIPI resin, as microwave polymerization involves agitation of the molecules and potential degradation during polymerization. This results in increased energy and temperature, which can lead to greater porosity in the material after monomer vaporization [59,62]. In addition, VIPI resin contains benzoyl peroxide, which can cause overheating during polymerization and create porosity in the material [63]. The decrease in porosity could be explained by water absorption during aging and the expansion of the sample size [64]. Additionally, the aging process may have removed the superficial layer of the samples, homogenizing the surface [64].

Regarding the relationship between microhardness and flexural strength, acrylic resin used for dental prostheses must have adequate flexural properties to resist masticatory forces and surface hardness to withstand abrasion. Both analyses assess resistance to plastic deformation. However, while flexural strength evaluates the structural flexibility of the material, hardness measures resistance to surface plastic deformation following mechanical indentation [65]. Although Group Diamond D did not fracture during flexural testing, the lower microhardness and higher flexibility may explain its ability to withstand more deflection, making it more ductile than the other groups. The results may be attributed to the polymerization process recommended by the manufacturer, which involves a short cycle with higher microwave power, leading to a higher number of residual monomers, reduced crosslink conversion, and fewer secondary polymerization reactions [66,67]. Although the composition and treatment of the added fibers are not disclosed by the manufacturer, the incorporation of fibers into the polymer matrix, when done optimally, can enhance the flexural strength of the resin [68]. On the other hand, another study evaluating Diamond D resin reported flexural strengths of 84.9 MPa before and 88.3 MPa after fatigue testing, which were statistically similar to the other PMMA materials evaluated in the study [69].

The resin used in provisional implant-supported complete fixed prostheses must be relatively flexible to resist fracture under masticatory forces [68]. However, it is important that these prostheses distribute the forces evenly across the implants, preventing overloading, especially when provisional prostheses are used while waiting for all implants to osseointegrate before proceeding with definitive rehabilitation [70,71]. In this context, a resin with a high deflection modulus may not be able to effectively distribute the forces [68].

According to ISO 20795-1:2013 [41], denture base materials must have a flexural strength of at least 65 MPa. Although no significant differences were found between Groups VIPI and VIPI + Wire, both groups exhibited flexural strength values above this threshold. With aging, the values decreased numerically but remained above the recommended limit. In addition, there was a decrease in porosity and an increase in roughness, likely due to factors mentioned earlier, such as water sorption during thermocycling [64] and a weakening of the polymer matrix [50,58].

The addition of orthodontic wire to VIPI resin did not improve flexural strength. From a clinical standpoint, wire-free provisional prostheses may offer greater ease of production. However, caution should be exercised when extrapolating in vitro results to clinical practice, as the shape and conditions of the tested samples differ from those of actual prostheses.

As a limitation of the study, the samples were not designed to simulate a real prosthesis, which limits the ability to extrapolate the results to clinical conditions. Polydispersity and molecular weight were also not evaluated in the current study. Additionally, future studies are recommended to explore complementary methodologies, such as cross-sectional analysis, atomic force microscopy (AFM), or brushing simulation, to strengthen the findings. Another limitation is related to the technologies used to fabricate the samples, such as digital technologies (3D printing and CAD/CAM), which were not included.

## 5. Conclusions

Although Group Diamond D did not fracture under flexural testing, it showed lower microhardness, higher roughness, and greater porosity than the other groups after aging. From this in vitro analysis, it is possible to conclude that Diamond D resin does not present adequate properties for use in provisional implant-supported full-arch prostheses. In contrast, VIPI with metallic reinforcement exhibited higher hardness, lower roughness, greater stability after aging, and reduced porosity.

## Figures and Tables

**Table 1:** Two-way repeated measures ANOVA for the microhardness, roughness, percentage of porosity per area, and pore size, and two-way ANOVA for the flexural strength of the resins tested, before and after thermocycling.

**Microhardness**	**SS**	**df**	**MS**	**F**	***p***
Resin	548.012	2	274.006	85.536	0.001 *
Among Samples	86.492	27	3.203		
Period	5.100	1	5.100	0.653	0.426
Resin × Period	40.499	2	20.250	2.591	0.093
Intra Samples	210.996	27	7.815		
**Roughness**	**SS**	**df**	**MS**	**F**	***p***
Resin	0.075	2	0.037	5.765	0.008 *
Among Samples	0.175	27	0.006		
Period	0.056	1	0.056	36.831	<0.001 *
Resin × Period	0.011	2	0.006	3.701	0.038 *
Intra Samples	210.996	27	7.815		
**Percentage of porosity per area**	**SS**	**df**	**MS**	**F**	***p***
Resin	1.713	2	0.856	11.361	<0.001 *
Among Samples	2.035	27	0.075		
Period	3.328	1	3.328	38.355	<0.001 *
Resin × Period	3.041	2	1.520	17.524	<0.001 *
Intra Samples	2.342	27	0.087		
**Pore size**	**SS**	**df**	**MS**	**F**	***p***
Resin	874.777	2	437.389	0.932	0.406
Among Samples	12,673.415	27	469.387		
Period	28,683.382	1	28,683.382	71.565	<0.001 *
Resin × Period	1003.998	2	501.999	1.252	0.302
Intra Samples	10,821.688	27	400.803		
**Flexural strength**	**SS**	**df**	**MS**	**F**	***p***
Resin	263.769	1	263.769	1.959	0.170
Period	151.224	1	151.224	1.123	0.296
Resin × Period	15.399	2	15.399	114	0.737
Error	4847.580	36	134.655		
Total	297,729.920	40	400.803		

* *p* < 0.05 denotes significant statistical difference.

## Data Availability

The original contributions presented in this study are included in the article. Further inquiries can be directed to the corresponding author.
